# Enabling Advanced Multi-Modal Neuroimaging Analysis within a Trusted Research Environment

**DOI:** 10.23889/ijpds.v9i5.1644

**Published:** 2024-09-18

**Authors:** Lewis Hotchkiss, Emma Squires, John Gallacher, Mark Newbury, Catrin Morris, Ronan Lyons, Simon Thompson

**Affiliations:** 1 Swansea University; 2 Oxford University

## Introduction

Globally, 55 million individuals have dementia, with an increasing annual incident of 10 million. Enabling the development of new multi-modal models can improve the current diagnostic pathways and potentially contribute to early diagnosis and treatment of dementia. Here, we report how multi-modal resources is achieved within the successful Trusted Research Environment (TRE).

## Objectives

We aimed to identify the challenges of the storage, distribution and analysis of neuroimaging data and how we could implement a comprehensive infrastructure to deal with these. The problems we specifically aimed to address were how to: anonymise scans, store large amounts of data, standardise datasets to a common format, extract metadata, provision the data, and allow for analysis. 

## Conclusion and Implications

We document various stages and capacities required for multi-modal neuroimaging analysis for dementia and conclude that achieving research ready assets to enable neuroimaging analysis for dementia from existing resources requires an engineered process to facilitate multiple aspects of curation, provisioning and large-scale analysis.

## Results

We developed an ingest pipeline for neuroimaging data to meet the requirements set out in the objectives. This involved standardising all datasets to the Brain Imaging Data Structure, defacing scans and anonymising data, using MinIO for data storage and extracting metadata from header information for data discovery and provisioning.

## Conclusion

The neuroimaging ingest pipeline developed has allowed for the distribution of imaging datasets within DPUK which has facilitated multi-modal research on anonymised and standardised data and enabled linkage with phenotypic and genomic data.

